# Secondary extramedullary plasmacytoma of sigmoid colon in a patient with multiple myeloma: a case report

**DOI:** 10.1186/s13256-018-1888-4

**Published:** 2018-12-25

**Authors:** Dimitris Fagkrezos, Konstantinos Manes, Konstantina Paraskeva, Michalis Lenos, Charikleia Triantopoulou, Dimitra Apessou, Petros Maniatis

**Affiliations:** 1 Computed Tomography Department, Konstantpouleio General Hospital Nea Ionia, Athens, Greece; 2 Surgery Department, Konstantpouleio General Hospital Nea Ionia, Athens, Greece; 3 Gastroenterology Department, Konstantpouleio General Hospital Nea Ionia, Athens, Greece; 4 Pathology Department, Konstantopouleio General Hospital Nea Ionia, Athens, Greece; 5 Radiology Department, Konstantpouleio General Hospital Nea Ionia, Athens, Greece

**Keywords:** Secondary extramedullary plasmacytoma, Sigmoid colon, Oncology, Multiple myeloma

## Abstract

**Background:**

Extramedullary plasmacytoma is an uncommon tumor that most often involves the nasopharynx or upper respiratory tract. Extramedullary plasmacytoma is a type of plasma cell neoplasm that can present as a primary tumor or secondary to another plasma cell neoplasm, such as multiple myeloma. Secondary extramedullary plasmacytoma is usually noted in the advanced stages of the disease. Involvement of the gastrointestinal tract occurs in approximately 10% of cases.

**Case presentation:**

A 71-year-old Caucasian woman with known diverticular disease of the colon and multiple myeloma diagnosed 3 years previously, with monoclonal bands of immunoglobulin A, lambda light chains, and multiple osteolytic lesions, presented to our hospital with abdominal pain, abdominal discomfort, and pneumoperitoneum. She underwent left colectomy for diverticulitis with perforation, and an extramedullary secondary colonic plasmacytoma was found in histopathological examination of the sigmoid colon.

**Conclusions:**

Plasmacytoma is known to occur in extraosseous sites. The stomach and small intestine are the most commonly involved sites in the gastrointestinal tract. Secondary extramedullary plasmacytoma of the colon is rare. Colonic plasmacytoma may have varying clinical presentations, such as inflammatory bowel disease and multiple colonic strictures. Although these cases are rare, treating physicians as well as radiologists, pathologists, and surgeons should be aware of this entity.

## Background

Plasma cell tumor is an immunoproliferative monoclonal disease of the B-cell line that originates from malignant transformed plasma cells. It is composed almost exclusively of plasma cells that are arranged in clusters or sheets with a scant, delicate, supportive connective tissue stroma. Plasmacytoma and multiple myeloma (MM) are two main groups of plasma cell tumors.

Plasmacytoma includes solitary plasmacytoma of bone and solitary extramedullary plasmacytoma (EMP). Secondary EMP has been reported rarely, and its natural history and diagnosis is not well defined. EMP of the colon is rare [1–3].

The presentation of gastrointestinal plasmacytoma is different from that occurring at other sites in the body. The most common presenting symptom is abdominal pain. Symptoms can include rectal bleeding, a change in bowel habits, weight loss, nausea, vomiting, large bowel obstruction, and intussusception [4]. They are generally visible as well-defined soft tissue masses on a computed tomographic (CT) scan and have heterogeneous enhancement on a magnetic resonance imaging (MRI) scan. Larger plasmacytomas can show aggressive traits such as invasion of adjacent fat, bone erosion, or vascular encasement. Positron emission tomography (PET) with ^18^F-labeled fluoro-2-deoxyglucose/computed tomography has also been found to be useful in staging and follow-up of EMP [5]. We report a case of secondary EMP of the sigmoid colon in a woman with known MM.

## Case presentation

A 71-year-old Caucasian woman with known diverticular disease of the colon and MM diagnosed 3 years previously, who was receiving chemotherapy with melphalan, bortezomib (a proteasome inhibitor), and a moderate dose of dexamethasone, presented to the emergency department of our hospital with abdominal pain, abdominal discomfort, and pneumoperitoneum. The clinical examination revealed acute abdomen with free air on an abdominal plain x-ray, and elevated levels of leukocyte cells at 18,000/μl and C-reactive protein at 145 mg/L. The patient had a family history free of cancer, and the last time she underwent colonoscopy was 1 year before admission. She had been diagnosed 3 years previously with stage II MM based on the International Staging System with a β_2_-microglobulin level of 4.2 mg/L and serum albumin level of 37 g/L. No study was performed for amyloidosis.

**Fig. 1:**
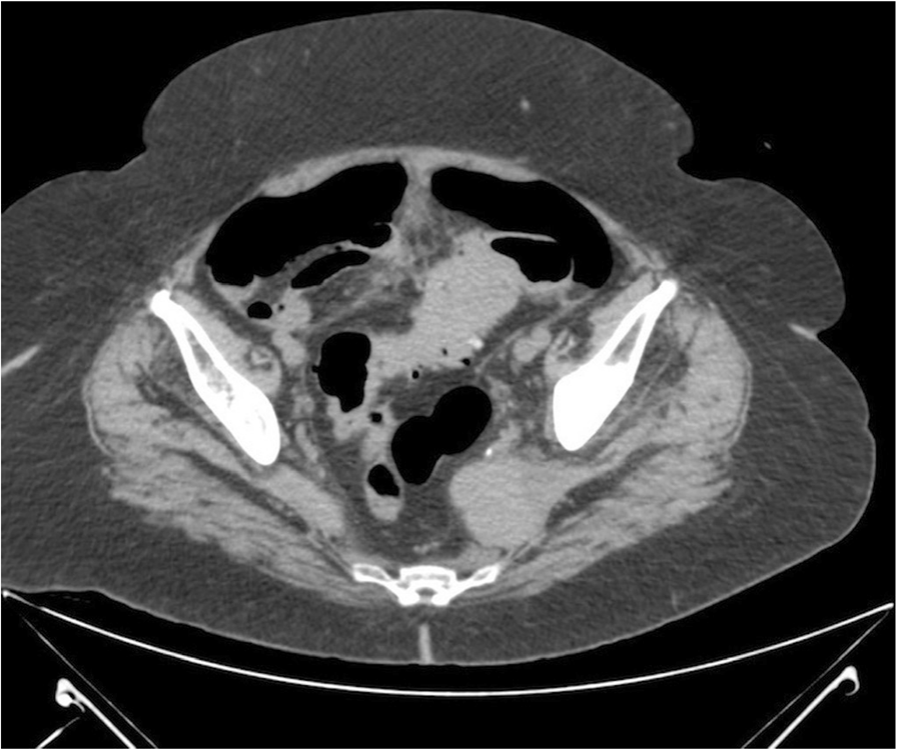
Axial computed tomography with no enhancement depicts a mass of soft tissue density in the anatomical area of the sigmoid colon and another compact lesion of soft tissue density on the left lateral wall of the minor pelvis. Presence of diverticula can be seen

**Fig. 2:**
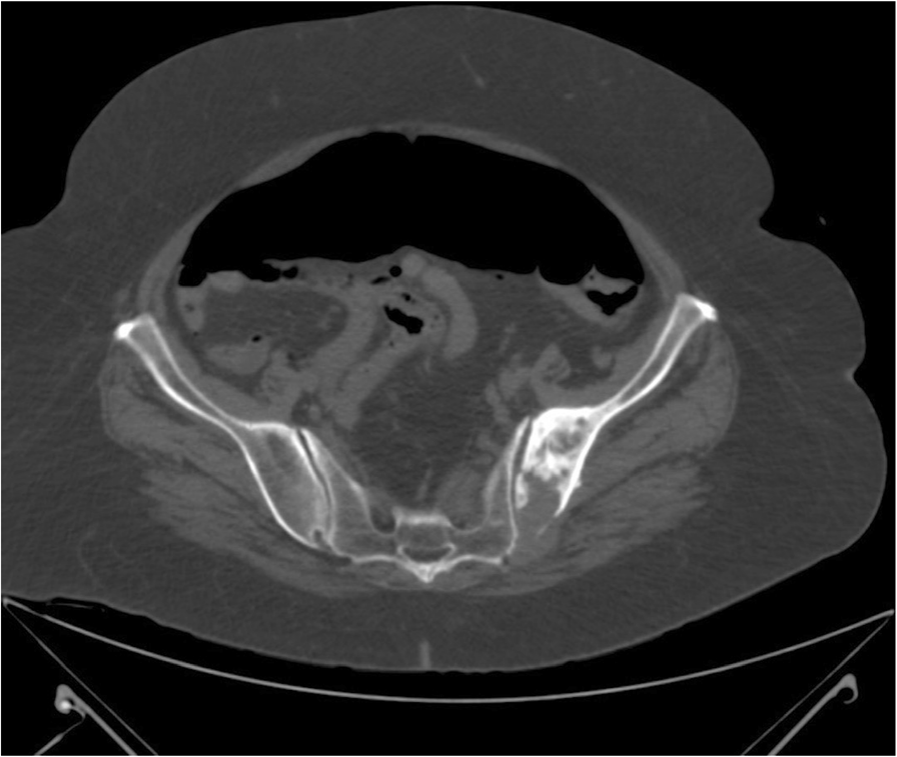
Pneumoperitoneum and osteolytic lesion on the left side of the pelvis with osteosclerotic component

**Fig. 3:**
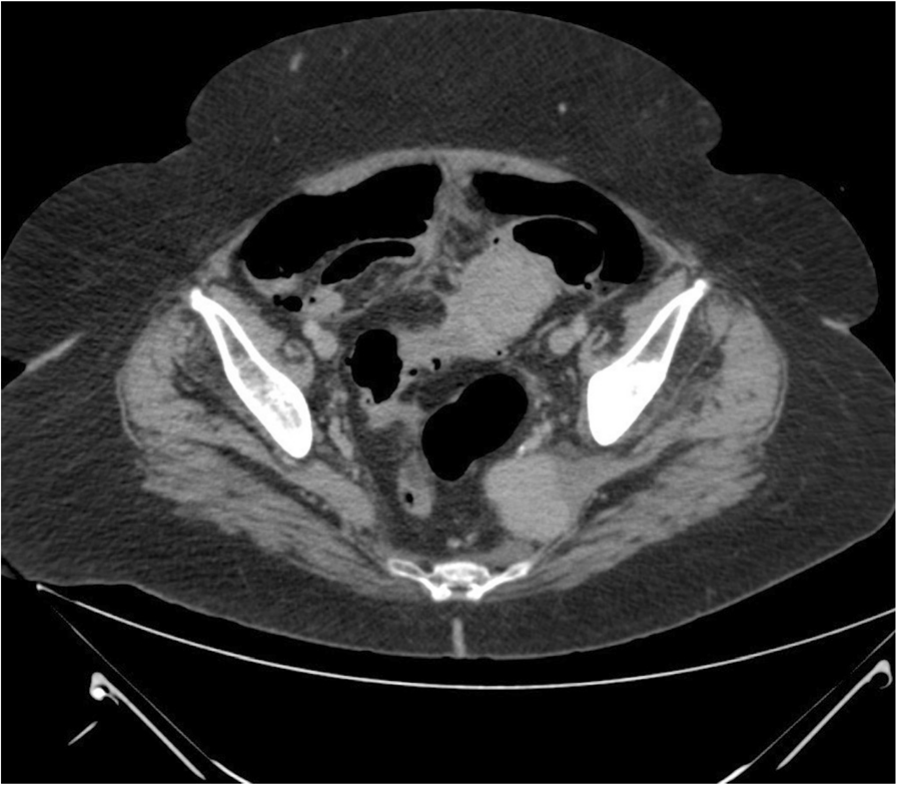
Axial computed tomography after administration of intravenous contrast shows enhancement of the lesions

**Fig. 4:**
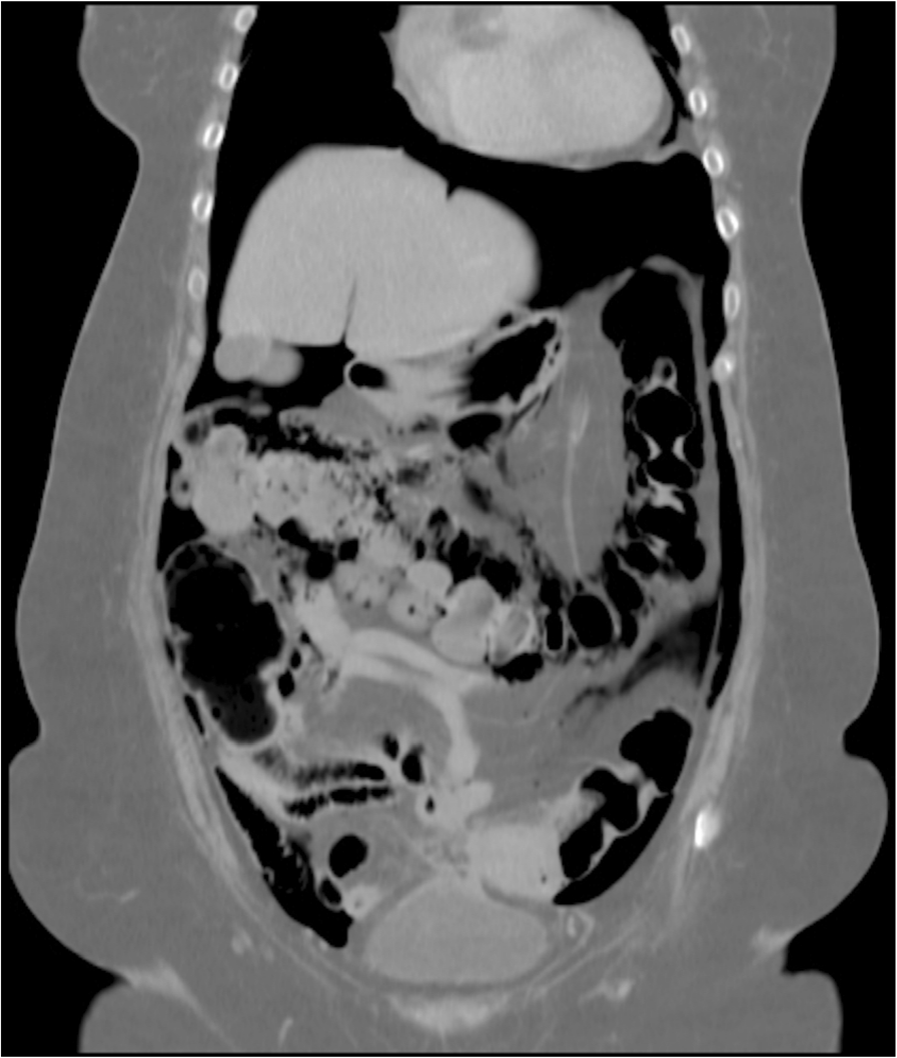
Coronal computed tomography of the distended colon and pneumoperitoneum

**Fig. 5:**
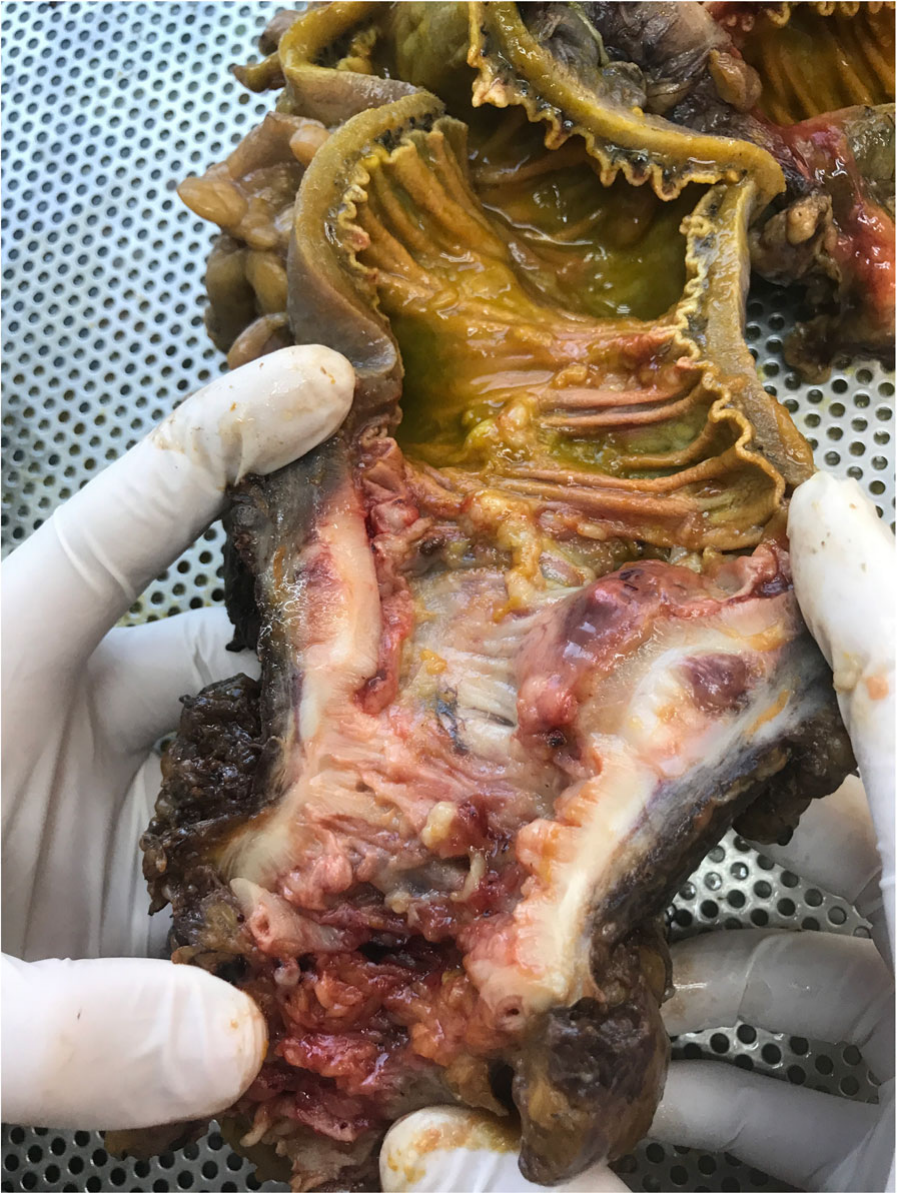
Surgical specimen of extramedullary plasmacytoma in the sigmoid colon

An abdominal CT scan showed a 5-cm-wide, 12-cm-long sigmoid mass with medial extension into the fatty tissue at the same localization, pneumoperitoneum, diverticula, free abdominal fluid collections, and distended loops of the small and large intestines (Figs. 1 and 2). The patient was urgently admitted to the operating room as soon as the diagnosis of colonic perforation was established. An exploratory laparotomy was performed, with supra- and subumbilical midline incisions. Intraoperative findings were purulent peritonitis and an obstructive mass of the sigmoid colon causing massive central colonic distention (Figs. 3 and 4). The mass was extending toward the mesosigmoid. It was impossible to define if the mass might be originating from the mesocolon (Fig. 5). After washing the whole abdomen meticulously, exploration revealed colonic perforation in the midtransverse colon at the mesocolic site. The mesocolon at this part was extremely inflammatory and had signs of venous congestion. An extended left colectomy was decided as the operation of choice for this patient. The operation was undertaken in an ontological fashion, with high ligation of the superior mesenteric artery and retrieval of all regional and apical lymph nodes. Peripheral transection was performed at the level of the upper rectum, and after removal of the specimen, a transverse stoma was established (a modified Hartmann’s procedure). Direct anastomosis was considered extremely risky owing to local inflammatory conditions (after peritonitis) and a highly distended colon (after obstruction).

**Fig. 6:**
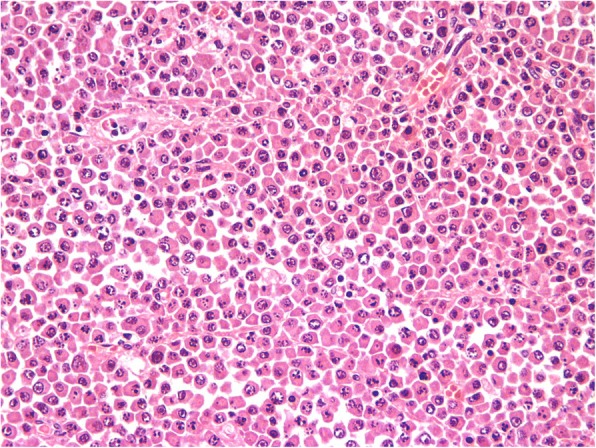
Microscopic evaluation (H&E stain, original magnification 400×). Large oval cells, consisting of a round nucleus in eccentric position with coarse chromatin, protruding nucleolus, and enough cytoplasm

**Fig. 7:**
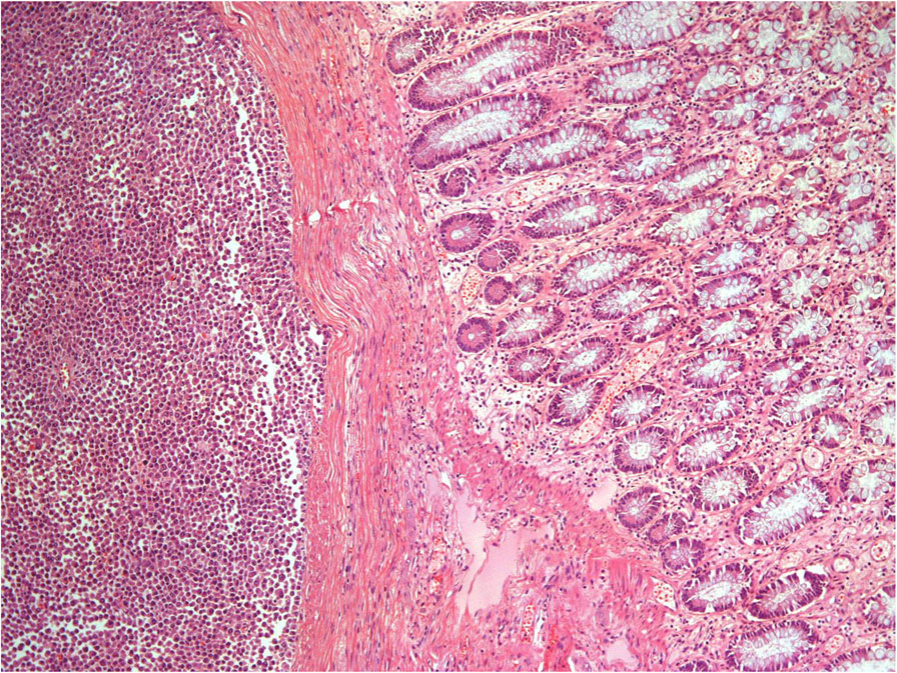
Microscopic evaluation (H&E stain, original magnification 100×). The neoplasm densely infiltrates the muscular wall of the intestine and the pericolic fat, without reaching the surface of the serum and without infiltrating the mucosa, which has severe ischemic lesions

**Fig. 8:**
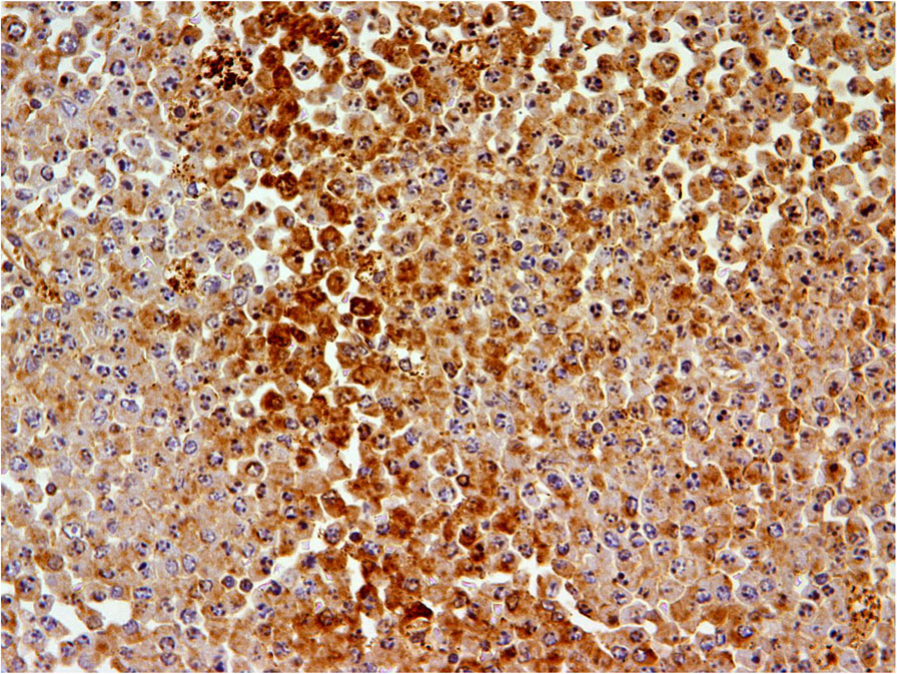
IHC analysis (CD138 stain, original magnification 400×) shows that the tumor cells are CD138^+^ with a few keratins

**Fig. 9:**
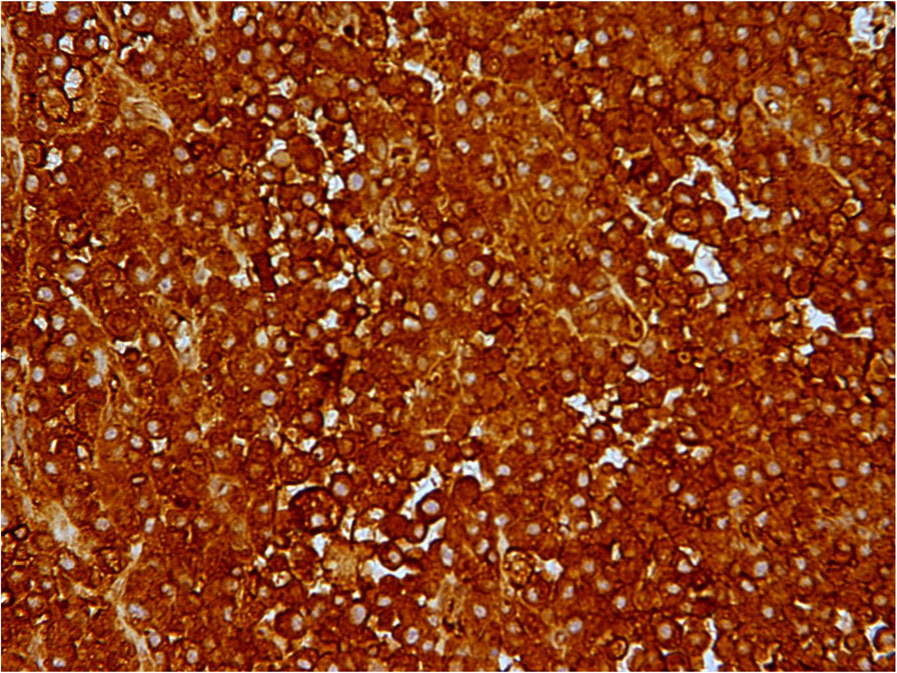
IHC analysis (light chain, original magnification 400×) for both kappa-light and lambda-light chains give an unreliable effect owing to a very high substrate material

The histopathologic study of the lesion showed large oval cells consisting of a round nucleus in eccentric position with coarse chromatin, protruding nucleolus, and enough cytoplasm (Fig. 6). There were also a few cells with double nuclei as well as giant nuclei. Few cells had the morphology of typical plasmacytes. There were many mitoses. Higher cell apoptosis was observed in the largest part of the tumor. The neoplasm densely infiltrated the muscular wall of the intestine and the pericolic fat without reaching the surface of the serum and without infiltrating the mucosa, which had severe ischemic lesions (Fig. 7). The surgical margins were free. In IHC analysis, the tumor cells were CD20^−^, CD5^−^, CD79a^+^, CD138^+^ (moderate), CD38^+^ (moderate), CD56^+^, CD10^−^, CD30^−^, CD117^+^ (a few), keratins AE1/AE3^−^, and S-100^−^ (Fig. 8). IHC analysis for both kappa-light and lambda-light chains gave an unreliable effect owing to a very high substrate material (Fig. 9). The Ki-67 cell proliferation index was approximately 40%. In the lymph nodes, morphologically and with CD138 and CD38 immunostaining in selected sections, infiltration by the neoplastic cell population was not documented. The patient did well postoperatively.

## Discussion

Hematological malignancies form a diverse group depending on their origin from bone marrow cells. They can be broadly classified as neoplasms of myeloid cell lines, neoplasms of lymphoid cell lines, lymphoproliferative disorders, and histiocytic/dendritic cell neoplasms.

Plasma cell neoplasm (PCN) is a type of hematological malignancy arising as a result of monoclonal proliferation of plasma cells (lymphoid cell line) and is associated with production of monoclonal proteins. These malignancies include monoclonal gammopathy of undetermined significance, solitary plasmacytoma of the bone, EMP, and MM.

EMP is a type of PCN that can present as a primary tumor or secondary to another PCN, such as MM. In the primary subtype, it occurs alone in the absence of another PCN, unlike in the secondary subtype, often with MM. MM is a monoclonal, immunoproliferative plasma cell neoplasm of the B-lymphoid cells. Secondary EMP is usually noted in the advanced stages of the disease. Primary and secondary EMPs mainly affect the upper aerodigestive tract (nasopharynx, oropharynx, hypopharynx, larynx, trachea, and esophagus).

EMP represents approximately 3% of all PCNs and occurs when malignant plasmablastic clones migrate into soft tissues. Plasmacytomas, first described by Schridde in 1905, are clonal proliferations of plasma cells that are cytologically and immunophenotypically identical to plasma cell myeloma but manifest a localized osseous or extraosseous growth pattern [6]. They are rare diseases, and the understanding of their epidemiologic features and clinical outcomes is derived largely from compilations of cases reported in the literature and in small clinical series [7].

The pathophysiological mechanism of EMPs is not yet fully determined. One suggestion is that MM cells and the bone marrow microenvironment tightly interact with each other. This is mediated by multiple soluble factors and adhesion molecules. Integrins acting as receptors on stromal cells and the extracellular matrix play an important role in the homing of MM cells in the bone marrow [8]. Changes in these adhesion molecules and integrins can lead to the migration of MM cells to the bloodstream (plasma cell escape) and finally to the development of an extraosseous plasmacytoma. In addition, in order for plasma cells to migrate outside the bone marrow, genetic changes must occur that give these migrating plasma cells their aggressive nature. Recent studies have investigated the expression of several glycoproteins in MM. Mutations in genes encoding these proteins, such as P53, play a major role in the aggressive character of the disease. P53 is a nuclear protein that provides genomic stability by regulating the cell cycle. Another protein, CD56, is usually present as a membrane glycoprotein on natural killer cells and T cells and is involved in cell adhesion and migration. CD56 is expressed in more than 70% of plasma cells in MM; however, it is not expressed in normal plasma cells. There is evidence that CD56 is downregulated in extramedullary MM.

Three-fourths of EMP cases are in males. EMP progresses to MM within 10 years in 11–30% of patients. Overall survival in 10 years is 70%. The median age of patients with either solitary bone plasmacytoma or EMP is 55 years. This median age is 10 years younger than that of patients with MM. EMP presents as a mass growing in the aerodigestive tract in 80–90% of patients, often with spread to lymph nodes, although other sites are affected as well.

Common complaints include swelling, headache, nasal discharge, epistaxis, nasal obstruction, sore throat, hoarseness, dysphonia, dysphagia, dyspnea, epigastric pain, and hemoptysis [9–12]. Symptoms arising from EMP in other tissues are associated with the site of the tumor, tumor size, and compression and/or involvement of the surrounding structures.

EMP involving the lung most commonly presents as a pulmonary nodule or hilar mass. Although EMP can occur in any site, 80–90% of tumors develop in the head and neck area, especially in the aerodigestive tract. Approximately 80% of cases involve the paranasal sinuses, pharynx, nasal cavity, or gums and oral mucosa [9, 12–15]. A mass (plasmacytoma) in these areas is the most common finding, with compression or invasion of the surrounding structures. Patients with tumors involving the base of the skull may present with cranial nerve palsies. Case reports of involvement of the urinary bladder, central nervous system, orbit, gastrointestinal tract (GIT), liver, spleen, pancreas, lung, breast, skin, testis, parotid gland, mediastinum, and thyroid gland (associated with goiter and Hashimoto thyroiditis) exist in the literature [9, 12–16].

In 30–40% of cases, local lymph nodes are involved at presentation or upon relapse. Because of the presentation of EMP in the mucosa of the aerodigestive tract (> 80%), the etiology may be related to chronic stimulation of inhaled irritants or viral infection. CT scanning, MRI, and complete endoscopic examination of the aerodigestive and gastrointestinal tracts are required to determine the exact extent of the tumor and its potential for resectability [14]. These lesions may be associated with variable mass effect; infiltration and/or destruction of adjacent bone, muscle, and fat; and vascular encasement [17].

EMP biopsy of the soft tissue lesion shows infiltration by monoclonal plasma cells. In EMP, the soft tissue lesion commonly exhibits submucosal growth, requiring deep biopsy, open biopsy, or complete excision, depending on the tumor location [14].

Histologically, EMP may be classified as low, intermediate, or high grade [18]. Bone marrow biopsy shows less than 5% plasma cells without evidence of clonality [9].

Prognosis and long-term follow-up of primary isolated EMP in the colon are not well known. Gupta *et al.* reported no recurrent tumor at 17-month follow-up in a case of EMP invading all the layers of the colon with metastases in three lymph nodes after postoperative adjuvant chemotherapy [4]. However, Doki *et al*. reported a recurrent case 4 months after operation [19]. Chemotherapy was not effective in that case; the patient died just 6 months after the tumor relapse.

The prognosis of primary EMP is generally good. Liebross *et al*. reported that median survival of 9.5 years, and 56% of patients were free from systemic disease at 5 years [1]. One-third of patients with EMP had MM after a median follow-up of 1.8 years.

Development of visceral secondary EMP involving the GIT in the course of MM (a monoclonal, immunoproliferative PCN of the B-lymphoid cells) is extremely rare, estimated to occur in 0.9% of cases [20]. Only 17 cases of EMP affecting the ileum have been reported in the English-language literature, almost all primary in nature [15], with only three previous cases of EMP secondary to MM reported [21–23].

Our patient was receiving chemotherapy for MM. In general, EMPs are considered to be radiosensitive, with a local control rate of 90–100% [7]. Allison *et al.* proposed surgery, radiotherapy (RT), and chemotherapy for gastrointestinal plasmacytomas according to the degree of disease extent [24].

Colonic plasmacytoma occurs only rarely, and a definitive algorithm for treatment has not been determined. Endoscopic treatments such as submucosal resection or polypectomy have proven to be sufficient in selected cases [25–27]. RT is the preferred treatment for anal canal and rectal lesions [28, 29]. An extensive review of over 400 publications done by Alexiou *et al*. provided evidence that surgery alone gives the best results in cases of solitary plasmacytoma when resectability is good. However, if complete surgical tumor resection is doubtful or impossible and/or if lymph node areas are affected, then combined therapy (surgery and radiation) is recommended [15]. In one recent series with 80 patients, younger patients, especially those with head and neck lesions and without pre-RT macroscopic tumors, seemed to have the best outcome when treated with RT, either with or without surgery [30].

In only one randomized trial of chemotherapy did patients receiving melphalan and prednisone show an improved disease-free survival rate [31]. The use of adjuvant chemotherapy is recommended for patients with higher-grade disease, local treatment failure (tumor size > 5 cm), or refractory disease [7].

Patients with EMP characteristically present with localized disease, and the incidence of lymph node involvement is 10–20%. The overall 10+-year survival rate is 70% [18]. Survival rates range from more than 90% among patients with EMP arising in the skin or lymph nodes to 48% for those with eye, brain, and central nervous system tumors [32]. Progression to MM varies from 10% to 30% in EMP. In a series with 258 patients (52 with EMP), bone localization was found to be the only predictor of MM development in multivariate analyses [33]. When progression to MM occurs, it usually does so within the first 2 years. Follow-up radiological and electrophoresis assessments are required after treatment to detect recurrence and progression to MM [18].

EMPs are rarely seen in the GIT. It is important to consider this possibility during the evaluation of a mass in the GIT because placmacytomas of GIT have different treatment and follow-up modalities than adenocarcinomas and other tumors of the GIT. Surgical treatment is usually sufficient in localized solitary plasmacytoma of the GIT.

In cases of secondary EMP, radiotherapy or surgery alone is insufficient, and systemic therapy is required. In younger patients, the use of immunomodulatory drugs with induction agents in combination with autologous hematopoietic stem cell transplant results in higher remission rates and overall survival [34]. The prognosis of secondary EMP with gastrointestinal involvement is unknown. However, the small number of cases reported in the literature indicates a poor prognosis [21–23, 34–36]. The role of surgery in secondary EMP is often palliative, to deal with resolvable life-threatening emergencies, and, where possible, to prolong life.

The prognosis of intestinal EMP is unclear. In nine selected patients with MM-associated EMPs, Vaiopoulos *et al.* [35] reported a median survival of 4.6 years, and none of the patients had intestinal involvement. Two cases involving the intestinal tract presented by Griffiths *et al.* had fatal outcomes not long after diagnosis with EMP [36].

## Conclusions

Because secondary EMP in the colon is rare, the clinical course, treatment guidelines, and prognosis are not very well defined. To provide adequate treatment, further study of the clinical features of secondary EMP of the colon is necessary.

## Abbreviations

CT: Computed tomography
EMP: Extramedullary plasmacytoma
GIT: Gastrointestinal tract
MM: Multiple myeloma
MRI: Magnetic resonance imaging
PCN: Plasma cell neoplasm
RT: Radiotherapy
